# Supplementary material for: Phonon-enhanced photothermoelectric effect in SrTiO3 ultra-broadband photodetector
Source: Nat Commun. 2019 Jan 11;10:138. doi: 10.1038/s41467-018-07860-0 (PMC6329832; doi:10.1038/s41467-018-07860-0)
Supplement: Supplementary file 1 — Supplementary Information [file 41467_2018_7860_MOESM1_ESM.pdf]

Supplementary Information

Phonon-enhanced photothermoelectric effect in strontium titanate ultra-broadband photodetector

Lu et al.

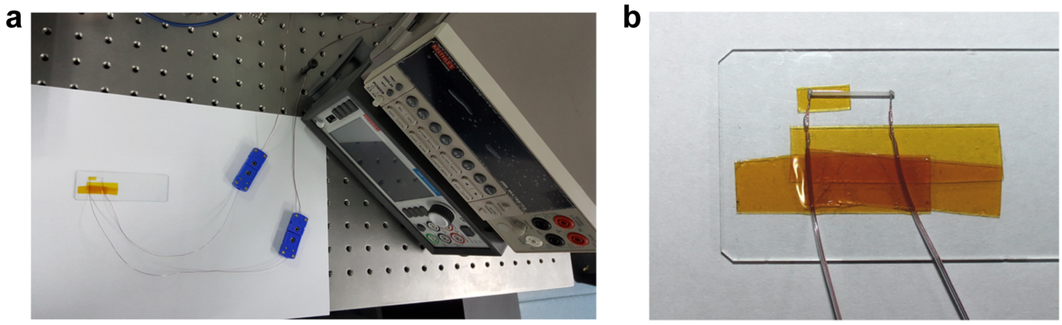

Supplementary Figure 1 Photograph of the setup. **a** Picture of the half-supported $SrTiO_3$ (STO) photodetector and the measurement setup. Keithley 2001 digital multimeter (DMM) and 2450 source meter are used to measure the photoresponse. **b** Enlargement of (**a**) to show the details. The STO crystal is 10 mm in length, 0.5 mm in width and 0.15 mm in thickness. Two pairs of T-type thermocouples are anchored to the two ends of STO crystal using silver paint. The left side of STO is glued on glass slide using the yellow double sided Kapton tape.

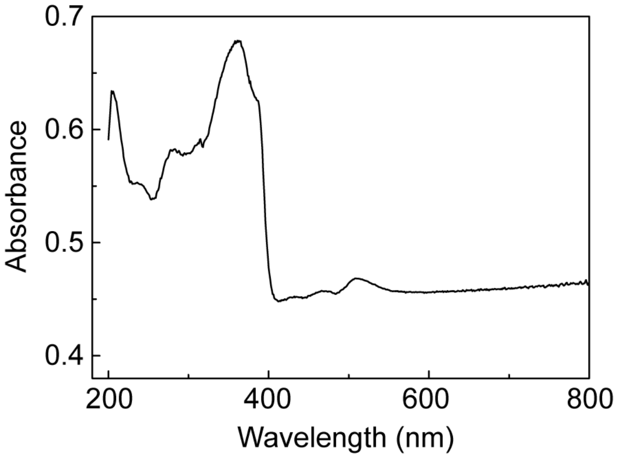

Supplementary Figure 2 Band gap determination. Ultraviolet-visible-near infrared spectrum of reduced $SrTiO_3$ (r-STO) annealed at 900 °C for 4h under H_2 atmosphere. The band gap of the r-STO crystal is about 3.1 eV.

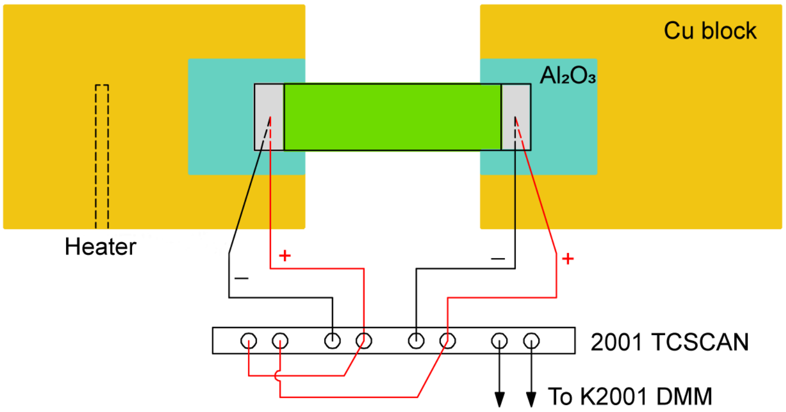

Supplementary Figure 3 Seebeck coefficient measurement. Schematic setup for the Seebeck coefficient measurement. The cuboid-shaped STO crystal is across two copper blocks. The cartridge heater inserted into one copper block is utilized to build a temperature gradient along the length of STO crystal. Two insulating aluminum oxide (Al_2O_3) crystals are used to electrically isolate the STO crystal from the underlying Cu blocks. Two pairs of T-type thermocouples are anchored to the two ends of STO crystal using silver paint. Keithley 2001 DMM equipped with a 2001-TCSCAN scanner card is used to record the voltage output as well as the temperature variations

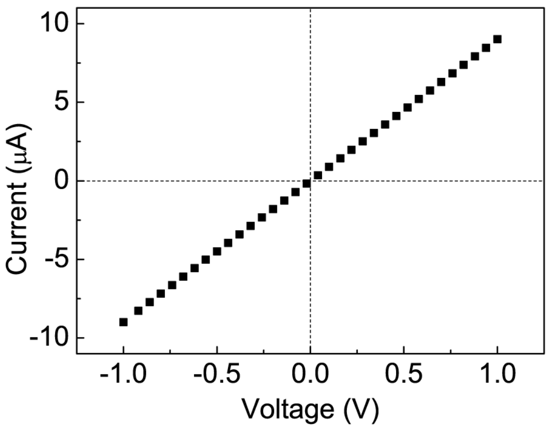

Supplementary Figure 4 Current-voltage relationship. Dark current-voltage (*I*-*V*) curve of r-STO with source voltage applied from -1 to 1 V. The linear relationship indicates an Ohmic contact is formed between r-STO and silver paint.

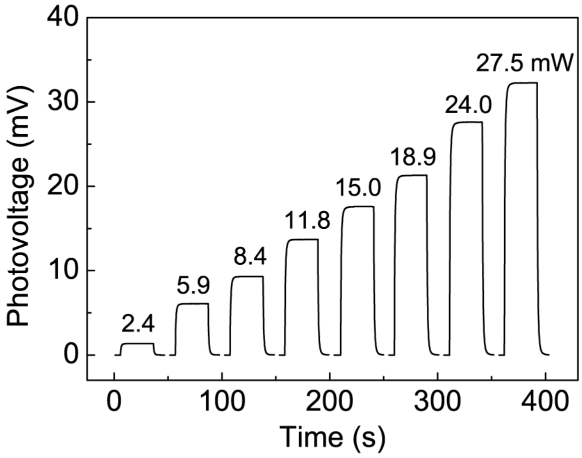

Supplementary Figure 5 Dynamic response. Response signals of r-STO detector under different laser powers (labelled on the top of each column). The illumination wavelength is 10.57 μm.

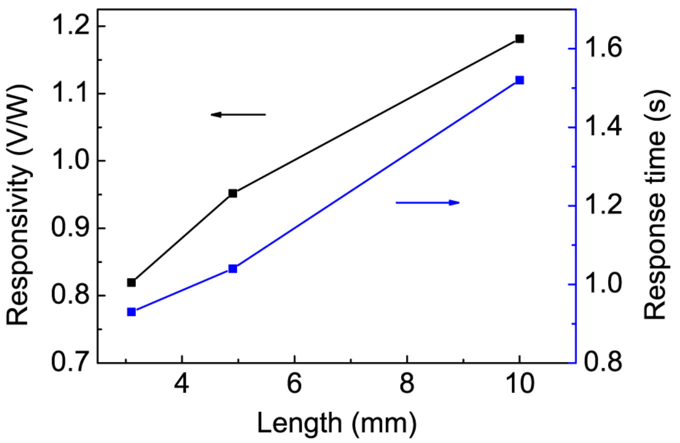

Supplementary Figure 6 Effects of $SrTiO_3$ channel length. Responsivity and response time as a function of STO length. The illumination wavelength is 10.57 μm, and the laser spot is placed on the right STO/Ag interface.

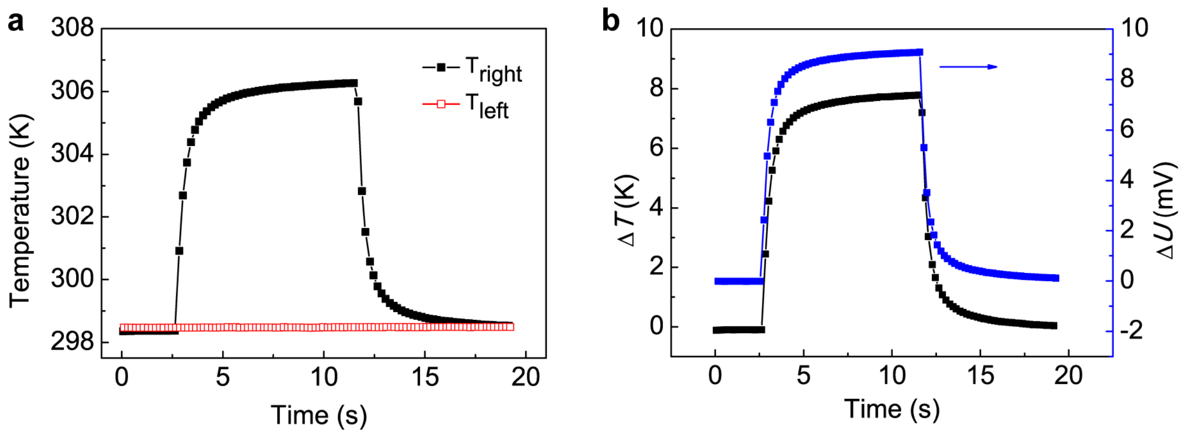

Supplementary Figure 7 Temperature evolution of fully supported r-STO detector. **a** Temperature responses of the two ends of r-STO crystal with laser illuminating the right STO/Ag interface. **b** Time dependent temperature difference and the corresponding photovoltage across the STO crystal. Illumination wavelength: 10.57 μm. Laser power: ~11.6 mW.

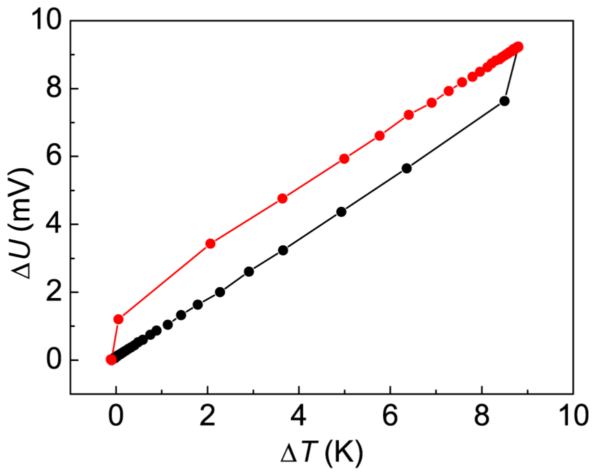

Supplementary Figure 8 Relationship between voltage output and temperature difference. Voltage output (Δ*U*) versus temperature difference (Δ*T*) with the laser spot located at STO crystal 500 μm away from the STO/Ag interface. The existence of the hysteresis-like behavior indicates this phenomenon is not related to the STO/Ag interface. The illumination wavelength is 10.57 μm.

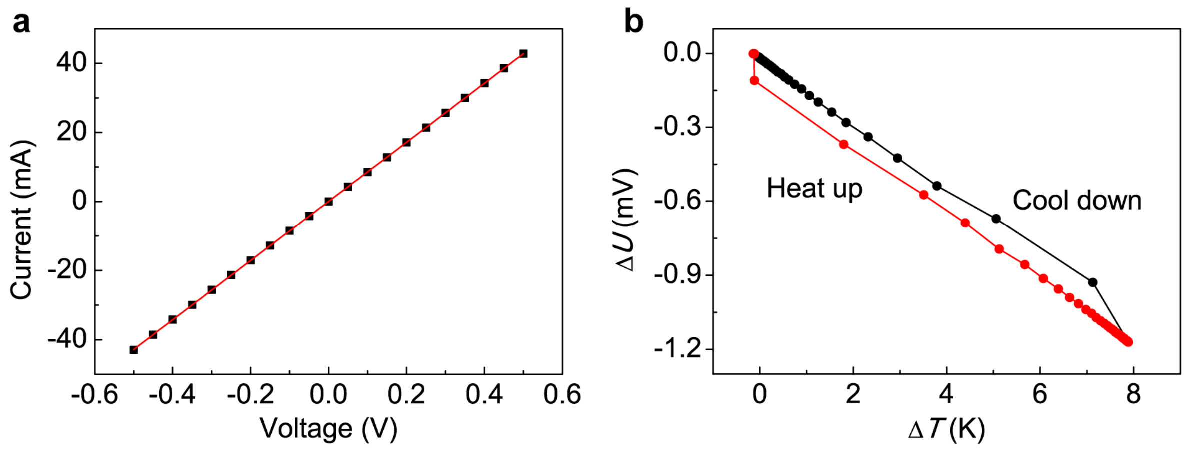

Supplementary Figure 9 Photothermoelctric response of MoS_2. **a** Dark *I-V* curve of 2 mol% vanadium-doped MoS_2. The red line is the linear fit to the data. **b** Voltage output (ΔU) versus temperature difference (ΔT) with the laser illuminating the MoS_2/Ag interface. MoS_2 size: 7 mm × 1 mm × 0.4 mm. Illumination wavelength: 10.57 μm. Laser power: 23 mW.

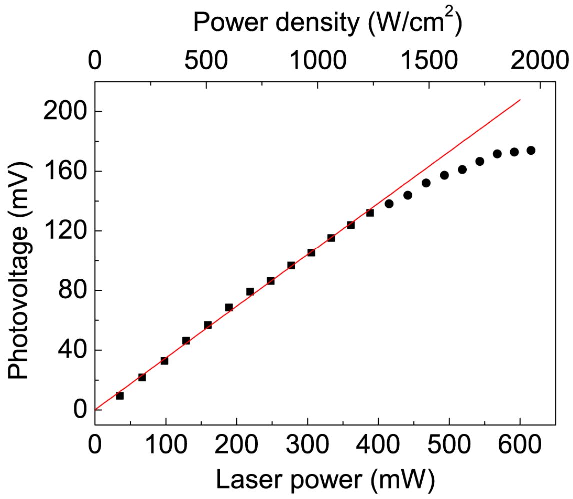

Supplementary Figure 10 Detection of high-power-density radiation. Photovoltage versus laser power of r-STO detector with fully supported configuration at λ = 1550 nm. The red line is the linear fit to the data from 0 to 388 mW.

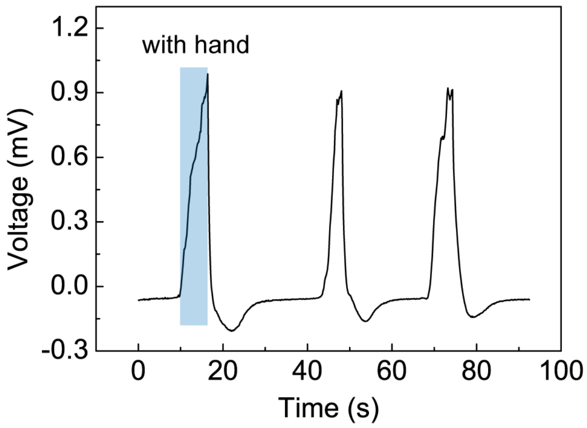

Supplementary Figure 11 Detection of low-power human radiation. Dynamic response with/without human figure placed above the suspended end of r-STO. The distance between the human figure and the detector is about 1.5 mm.

Supplementary Table 1 Performance parameters of some representative photothermoelectric detectors.

Active material	Reported response range	Responsivity	Response time	Ref.
Graphene/SiO_2 (phonon enhancement)	6.4 μm-10 μm	78 nA W^{-1}@9.3 μm	–	1
Graphene (biased; p-n junction)	400 nm-1600 nm	0.5 V W^{-1}@457 nm	–	2
Graphene (asymmetric electrodes)	1.54 μm; 119 μm	10 V W^{-1}@119 μm<br>0.25 V W^{-1}@1.54 μm	Sub-100 ps	3
Bi_2Se_3 nanoribbon	514 nm	2.6 V W^{-1}	0.7 s	4
Bi_2Te_3/Sb_2Te_3 wire array (plasmon enhancement)	500 nm-750 nm	38 V W^{-1}@646 nm	0.3 ms	5
Black phosphorus (biased)	1006 μm	0.15 V W^{-1}	–	6
MoS_2 (unbiased)	532 nm; 700 nm	1 V W^{-1}@700 nm	–	7
Nanoporous silicon	476 nm-514 nm	–	5 s	8
Carbon nanotube (suspended)	685 nm	0.3 V W^{-1}	17 ms	9
Carbon nanotube (p-n junction)	660 nm-3.3 μm	1 V W^{-1} @660 nm	0.6 s	10
Thorlab S175C power detector	190 nm-17 μm	0.17 V W^{-1}	40 s	–
Thorlab S302C power detector	185 nm-20 μm	0.3 V W^{-1}	3.6 s	–
This work	325 nm-10. 67 μm	1.18 V W^{-1}@10.57 μm	1.52 s	–

Supplementary References

1 Badioli, M. *et al.* Phonon-mediated mid-infrared photoresponse of graphene. *Nano Lett.* **14**, 6374-6381 (2014).

2 Echtermeyer, T. J. *et al.* Photothermoelectric and photoelectric contributions to light detection in metal–graphene–metal photodetectors. *Nano Lett.* **14**, 3733-3742 (2014).

3 Cai, X. *et al.* Sensitive room-temperature terahertz detection via the photothermoelectric effect in graphene. *Nat. Nanotechnol.* **9**, 814-819 (2014).

4 Yan, Y. *et al.* Topological surface state enhanced photothermoelectric effect in Bi_2Se_3 nanoribbons. *Nano Lett.* **14**, 4389-4394 (2014).

5 Mauser, K. W. *et al.* Resonant thermoelectric nanophotonics. *Nat. Nanotechnol.* **12**, 770-775 (2017).

6 Viti, L. *et al.* Black phosphorus terahertz photodetectors. *Adv. Mater.* **27**, 5567-5572 (2015).

7 Buscema, M. *et al.* Large and tunable photothermoelectric effect in single-layer MoS_2. *Nano Lett.* **13**, 358-363 (2013).

8 Lai, Y. S. *et al.* Photothermoelectric effects in nanoporous silicon. *Adv. Mater.* **28**, 2644-2648 (2016).

9 DeBorde, T., Aspitarte, L., Sharf, T., Kevek, J. W. & Minot, E. D. Photothermoelectric effect in suspended semiconducting carbon nanotubes. *ACS Nano* **8**, 216-221 (2013).

10 He, X. *et al.* Photothermoelectric p–n junction photodetector with intrinsic broadband polarimetry based on macroscopic carbon nanotube films. *ACS Nano* **7**, 7271-7277 (2013).
